# Keep it together: restraints in crystallographic refinement of macromolecule–ligand complexes

**DOI:** 10.1107/S2059798316017964

**Published:** 2017-02-01

**Authors:** Roberto A. Steiner, Julie A. Tucker

**Affiliations:** a Randall Division of Cell and Molecular Biophysics, King’s College London, London SE1 1UL, England; b Northern Institute for Cancer Research, Paul O’Gorman Building, Medical School, Newcastle University, Framlington Place, Newcastle-upon-Tyne NE2 4HH, England

**Keywords:** restraint sets, ligand complexes, standard deviation, macromolecular crystallography, refinement

## Abstract

A short introduction is provided to the concept of restraints in macromolecular crystallographic refinement. A typical ligand restraint-generation process is then described, covering types of input, the methodology and the mechanics behind the software in general terms, how this has evolved over recent years and what to look for in the output. Finally, the currently available restraint-generation software is compared, concluding with some thoughts for the future.

## Abstract 2

An overview of the process of ligand restraint generation for macromolecular crystallographic refinement is given.

## 1. Introduction   

The limited resolution at which macromolecular crystals typically diffract does not allow crystallographic refinement to be carried out using solely X-ray diffraction data. Prior knowledge, often in the form of stereochemical restraints, also needs to be taken into account to achieve chemically plausible structures (Evans, 2007 ▸). Macromolecular refinement packages thus minimize a target function with two components: a component utilizing geometry (or prior knowledge) and a component utilizing experimental X-ray knowledge,
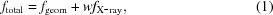
where *f*_total_ is the total target function to be minimized, consisting of functions controlling the geometry of the model (*f*_geom_) and the fit of the model parameters to the experimental data (*f*_X-ray_), and *w* is a weight between the relative contributions of these two components. Optimization routines are available in most packages that allow an automatic selection of *w*. From a Bayesian viewpoint, these functions have the following probabilistic interpretation:
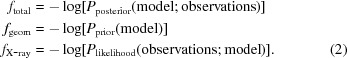
A number of research articles describe these functions in detail together with their implementation in the various refinement packages available as well as the mathematical tools to minimize *f*_total_. In the case of *REFMAC*5, the software provided with the *CCP*4 suite, the reader is encouraged to consult the following articles: Murshudov *et al.* (1997 ▸, 1999 ▸, 2011 ▸), Nicholls *et al.* (2012 ▸), Skubák *et al.* (2004 ▸, 2009 ▸), Steiner *et al.* (2003 ▸) and Vagin *et al.* (2004 ▸).

The term *f*_geom_ in (1) encodes specifically prior knowledge about the macromolecular system to be refined and is built of several components. These include the following.

(i) Stereochemical information (*e.g.* bond distances, angles) about the constituent blocks (*e.g.* amino acids, nucleic acids) of macromolecules and the covalent links between them.
(ii) The internal consistency of macromolecules (*e.g.* non-crystallographic symmetry, if present).
(iii) Additional structural knowledge (similarity to known structures, restraints on current interatomic distances or secondary-structure elements *etc.*).

A simple example of (i) is given by bond-distance information
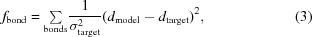
where *d*_model_ are the bond lengths calculated from the model and *d*_target_ and σ_target_ are the ‘ideal’ value of this particular geometric parameter and its standard deviation, respectively. Equations similar to (3) are also used for other stereochemical terms that collectively define *f*_geom_:
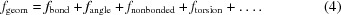
For protein refinement all major packages rely on the CSD-X library, a set of high-quality restraints introduced by Engh & Huber (1991 ▸) based on the small-molecule structures from the Cambridge Structural Database (CSD; Allen, 2002 ▸). More recently, however, the use of a conformation-dependent library (CDL), in which target values and standard deviations for protein main-chain bond lengths and angles vary as a function of the local φ/ψ angles, has been shown to improve refinement behaviour across the resolution range (Berkholz *et al.*, 2009 ▸; Tronrud *et al.*, 2010 ▸; Tronrud & Karplus, 2011 ▸). From the user’s perspective, the task of refinement is greatly simplified by the availability of these ‘libraries’ accessed by the refinement engines that effectively allow the definition of *f*_geom_ ‘on the fly’. The CCP4 monomer library (Vagin *et al.*, 2004 ▸), used by *REFMAC*5 and other packages including *phenix.refine* (Adams *et al.*, 2010 ▸), *Coot* (Emsley & Cowtan, 2004 ▸; Emsley *et al.*, 2010 ▸) and the *PDB_REDO* server (Joosten *et al.*, 2012 ▸, 2014 ▸), contains almost 13 500 monomers and more than 130 link/modification descriptions providing stereochemical knowledge for amino acids, nucleic acids and common small molecules such as enzyme cofactors and crystallization-solution components. The current version of the *phenix.refine* ‘dictionary’ also includes CDL restraints for the protein backbone (Moriarty *et al.*, 2016 ▸). Whilst macromolecular refinement often proceeds with virtually no manual intervention, user intervention is, however, still required when chemical components are encountered that are not present in the available libraries. Setting up restraints for these components can still pose a challenge for the novice (and occasionally even the expert) user.

At the time of writing, more than three quarters of the X-ray crystal structures deposited in the Worldwide Protein Data Bank (wwPDB; Berman *et al.*, 2003 ▸) contained one or more small molecules in addition to their macromolecular content. These may have been deliberately introduced by the experimenter as deemed to be functionally relevant, or be accidental arrivals having been co-purified with the macromolecular component or formed part of the crystallization/cryocooling solutions. They comprise a wide variety of chemistries, both natural and synthetic, ranging from co­factors, substrates and physiological ligands through to metal clusters, ions, solvent molecules, inhibitors and potential drugs. Dictionary-generation software exists to provide stereochemical restraints and, where required, starting coordinates for these novel molecules.

The subject of restraints on the small-molecule components of macromolecular structures was last reviewed in 2007 (Kleywegt, 2007 ▸). However, significant progress has been made over the intervening decade in the underlying method­ologies and automation of both starting-coordinate and restraint generation. This review will focus on these develop­ments, and we refer the reader to Kleywegt *et al.* (2003 ▸) and Kleywegt (2007 ▸) for historical perspectives.

## 2. The dictionary-generation process   

**Figure 1:**
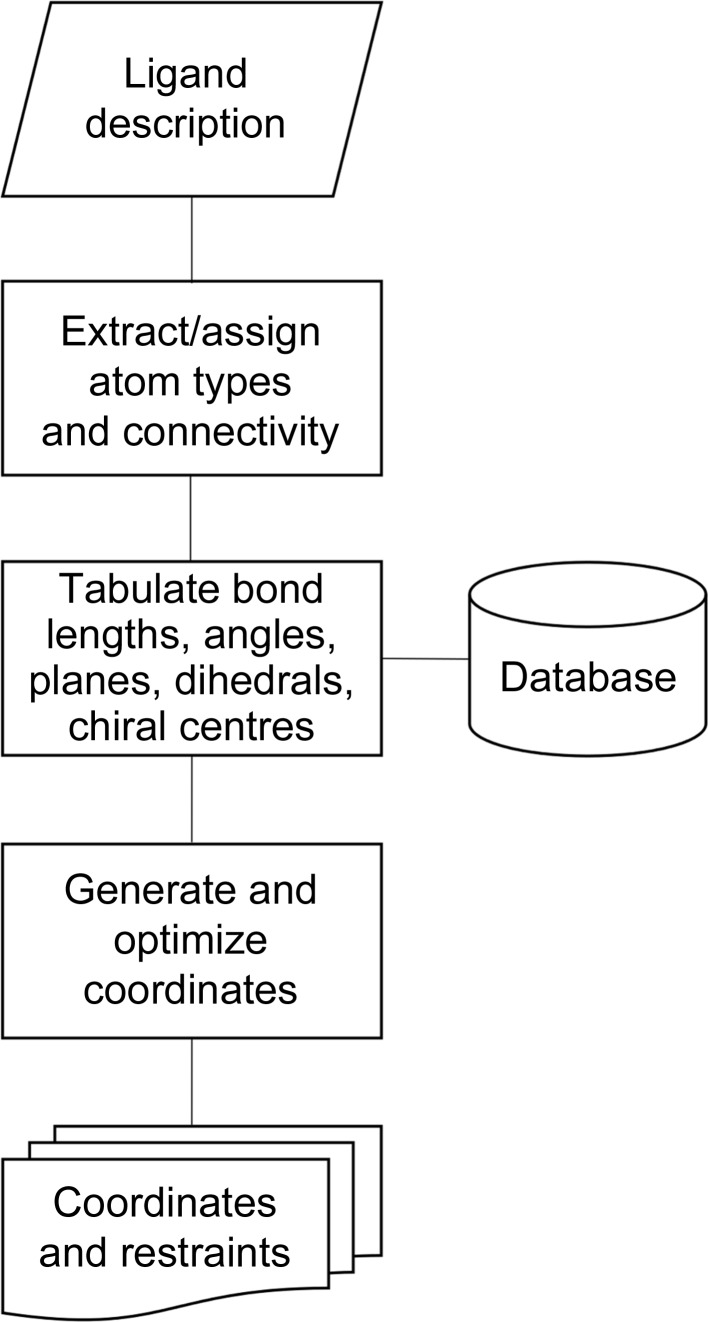
Schematic of the dictionary-generation process.

**Figure 2:**
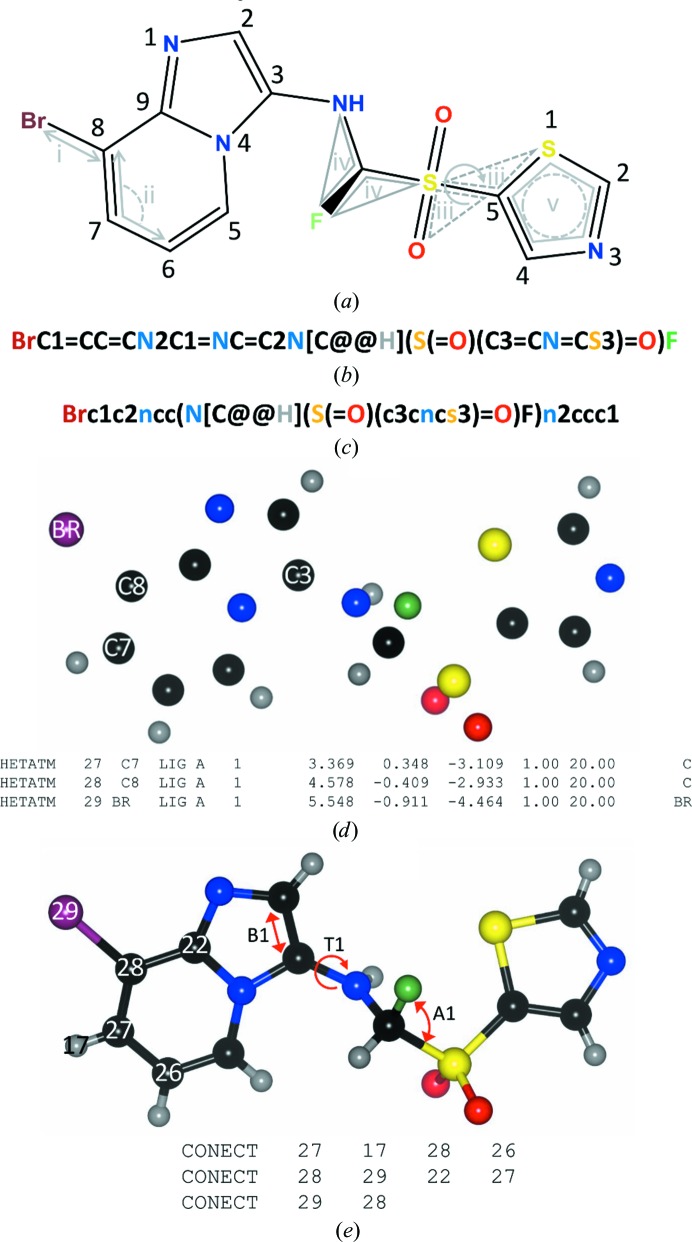
Types of input to a dictionary generator, illustrated using a hypothetical example molecule, chimerin1. Chimerin1 may be described using a two-dimensional sketch (*a*), as a SMILES string of different types (*b*, *c*) or as a set of coordinates, illustrated here in PDB format both without (*d*) and with (*e*) CONECT records. Restraint types are illustrated in (*a*): a bond-length restraint between two atoms (i), a bond-angle restraint between three bonded atoms (ii), a dihedral restraint relating four atoms (iii), a chiral restraint (iv) and a planar restraint (v). (*a*)–(*c*) were prepared using *ChemBioDraw Ultra* 14.0 (PerkinElmer) and (*d*) and (*e*) using *ACEDRG* (Long *et al.*, 2017 ▸) to generate coordinates and *CCP*4*mg* (McNicholas *et al.*, 2011 ▸) for rendering.

In general terms, the process of generating a set of restraints, or ‘dictionary’, for a small molecule involves (i) taking a description of the molecule as an input, (ii) processing its description to derive atom energy types and connectivities, and finally (iii) using this information to generate an idealized set of coordinates to allow fitting of the ligand to electron density and a list of geometric restraints with associated weights to allow the fitted ligand to be refined (Fig. 1 ▸). Each program uses different approaches to achieve these latter two steps and these will be covered in more detail in §[Sec sec3]3. Firstly, we will discuss the possible types of input to, and output from, a dictionary-generation program, and illustrate the importance of providing an appropriate molecular description. We will use a hypothetical molecule, which we have called chimerin1 (Fig. 2 ▸), to illustrate the principles of the dictionary-generation process.

### 2.1. Dictionary inputs and outputs   

Chimerin1, or to give it its full IUPAC name (*R*)-8-bromo-*N*-[fluoro(thiazol-5-ylsulfonyl)methyl]imidazo[1,2-*a*]pyridin-3-amine, can be described in a number of ways. Sketches are a fairly intuitive and easy depiction for a person to understand (Fig. 2 ▸ *a*); however, a more abbreviated format called a SMILES string (Weininger, 1988 ▸), or Simplified Molecular Input Line Entry System string, is a more compact and, importantly, both machine- and human-readable molecular descriptor (Figs. 2 ▸ *b* and 2 ▸ *c*). Both two-dimensional sketches and SMIILES strings can come in different ‘flavours’, however, and chimerin1 can be described in at least two non-equivalent ways, as illustrated by the two SMILES strings shown in Figs. 2 ▸(*b*) and 2 ▸(*c*). In Figs. 2 ▸(*a*) and 2 ▸(*b*), chimerin1 is represented in ‘Kekulized’ form with alternating single and double bonds, whilst in Fig. 2 ▸(*c*) chimerin1 is represented with the heterocycles as aromatic and delocalized. The definition of atom types (§3.1), and thus restraints and starting coordinates, can vary depending on which input representation is used.

In contrast to SMILES strings and two-dimensional sketches, a coordinate file can be a surprisingly ambiguous description of a molecule. In its simplest form, a coordinate file contains information on the name, coordinates (in the example used here these are in *xyz* Cartesian space), occupancy, atomic displacement parameters (*B* factors) and element type for each atom in the molecule of interest (Fig. 2 ▸ *d*). It does not explicitly define the connectivity between the atoms unless it is supplemented with CONECT records (Fig. 2 ▸ *e*). The coordinate file illustrated contains explicit H atoms; these help the dictionary-generation software to assign atom types, hybridization states and bond orders. All of this information must otherwise be inferred from the distances and angles between the atoms.

In summary, from the perspective of a dictionary generator, not all input files are equal. The *phenix.elbow* documentation captures this very succinctly:

where possible use a SMILES string or Chemical Components code (this is the three letter code for a molecule that is already present in the PDB, for example ATP). If you must use a PDB file make sure it contains explicit H atoms and CONECT records as automated topology determination is unreliable, and you may get back a different molecule than you were expecting

(Moriarty *et al.*, 2009 ▸). The Uniform Resource Locators (URLs) for *phenix.elbow* and other web resources mentioned in this article are provided in Supplementary Table S1.

Outputs can be equally varied, with restraints files variously known as dictionaries (molecule.dict), libraries (molecule.lib), crystallographic information files (molecule.cif) and topology and parameter files (molecule.toppar). The idealized coordinates may also be written in various formats, for example Protein Data Bank (molecule.pdb), Molfile (molecule.mol) and structure-data file (molecule.sdf).

## 3. How are restraints generated?   

Chimerin1 has 29 atoms, of which 21 are heavy atoms (*i.e.* non-H), and it can be described using 31 bonds, 51 angles, 19 dihedrals (or torsions), one chiral centre and at least two planar restraints. These restraint types are illustrated diagrammatically in Fig. 2 ▸(*a*). One could write out the restraints for chimerin1 by hand, and historically that is how dictionaries were constructed; however, as the size and complexity of a novel molecule increases, this rapidly becomes unmanageable. Even for a relatively small molecule getting the chemistry right can be nontrivial.

### 3.1. Atom energy types   

The first key step in generating a dictionary is to define what is called the ‘atom energy type’ for each atom in the molecule. The energy type of an atom is determined by the chemical element (carbon, nitrogen, oxygen, hydrogen, sulfur, bromine, fluorine *etc.*), and its connectivity within the network of atoms that comprise the molecule of interest. Hence the importance of supplying the dictionary generator with the richest possible input, although most programs do have methods to derive the required information from less optimal input. Table 1 ▸ shows for three atoms in chimerin1 how the atom energy types could be matched with definitions available in the CCP4 library of atom energy types, ener_lib.cif.

### 3.2. Experimental *versus* theoretical data sources   

Once atom energy types have been defined, these can be used to interrogate various sources of experimental information such as the wwPDB Chemical Components Dictionary (wwPDB CCD; Westbrook *et al.*, 2015 ▸), the CSD (Groom & Allen, 2014 ▸; Allen, 2002 ▸) or the Crystallography Open Database (COD; Gražulis *et al.*, 2009 ▸, 2012 ▸) to derive bond distances, bond angles and torsional restraints. Alternatively, where experimental data are lacking, a molecular-simulation approach can be used to calculate the various restraint parameters. Importantly, these approaches can be used to define both the ideal values for the various restraints in a molecule (*d*_target_ in equation 3) and their associated standard deviations (σ_target_ in equation 3).

Molecular-simulation approaches use a force-field function (5), which is similar to the refinement target function (1), and defines the energy of the molecule as a sum of terms describing the bonded and nonbonded interaction energies, which are then minimized:
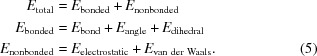
There are many different force fields, which use different forms for the various interactions within and between molecules, and the parameters of which are variously derived from experimental data, theoretical data or a combination of the two; details of the force fields that are most commonly used in ligand dictionary generation are given in Table 2 ▸. A key aspect of both the force-field form and the force-field parameters is that parameters for a particular atom or group of atoms should be the same for different molecules, *i.e.* they should be transferable. Without this property a different force field would be required for each and every new molecule. A similar notion of transferability applies to the use of experimental restraint information (Long *et al.*, 2017 ▸).

The methods and data sources used by current dictionary generators to derive restraints and standard deviations are summarized and compared in Table 3 ▸. The majority of these programs are freely available to academic users, and two (*PRODRG*2 and *grade*) are also available through web servers (see Supplementary Table S1 for URLs), obviating the need for a local installation.

In recent years, there has been a convergence towards the use of the CSD as a source of experimental restraints and their associated standard deviations. In general, small-molecule experimental data (extracted from the CSD) are used alongside a force-field approach, except in the case of *writedict*, where force fields are used exclusively to generate restraint information. Further details of the philosophy and method­ology underlying individual programs are available in the original references (Table 3 ▸) and will not, therefore, be covered here.

### 3.3. Comparing dictionary generators   

**Figure 3:**
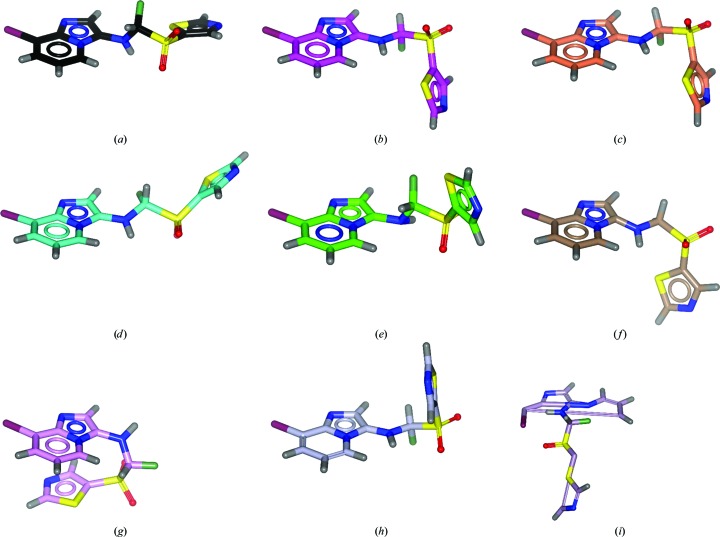
Comparison of output coordinates from selected dictionary generators: (*a*) *ACEDRG*, (*b*) *astex_prepare_dictionary*, (*c*) *Corina*, (*d*) *phenix.elbow*, (*e*) *grade*, (*f*) *PRODRG*2, (*g*) *Pyrogen*, (*h*) *writedict* and (*i*) *Libcheck*. Coordinates were overlaid using the Superpose Ligand function in *Coot* (Debreczeni & Emsley, 2012 ▸), with minor manual adjustment if required, and then displayed and rendered using *CCP*4*mg* (McNicholas *et al.*, 2011 ▸).

**Figure 4:**
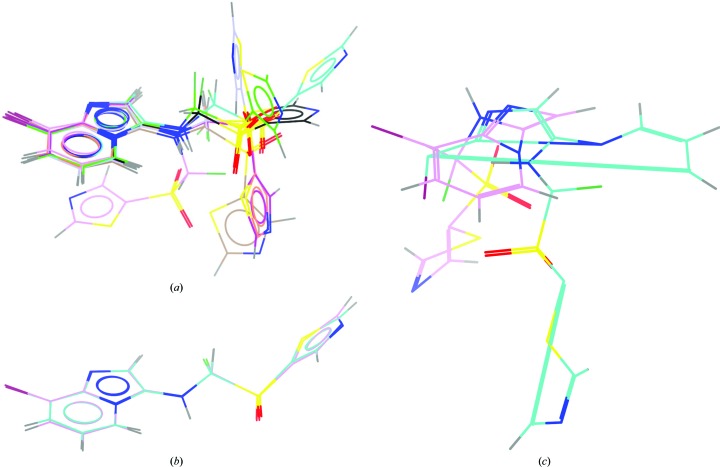
Comparison of output coordinates from selected dictionary generators (*a*, *c*) before and (*b*, *c*) after idealization. (*a*) Overlay of output coordinates from selected dictionary generators (Figs. 3 ▸ *a*–3 ▸ *h*), aligned and coloured as in Fig. 3 ▸. *Libcheck* (Fig. 3 ▸ *i*) has been omitted for the sake of clarity. Overlay of coordinates from (*b*) *phenix.elbow* and (*c*) *Libcheck* before (C atoms coloured cyan) and after (C atoms coloured pink) idealization in *REFMAC*5.

The performance of a range of dictionary generators was assessed by providing the chimerin1 SMILES string and, where possible, running *via* the command line using default parameters (§S1, Supporting Information). Output coordinates are shown in Fig. 3 ▸. With one exception (*Libcheck*; Fig. 3 ▸ *i*), all of the dictionary generators provide an acceptable starting point for further optimization. There are some differences in the assignment of aromaticity to the heterocyclic rings, and a wide variation in the torsion angles around the bond linking the imidazopyridine ring and the exocyclic amine group (labelled T1 in Fig. 2 ▸ *e*). This is particularly obvious when the output coordinate files are overlaid on the imidazopyridine ring (Fig. 4 ▸ *a*). In general, torsional variation in initial coordinates will not be problematic, as torsional conformation space will be sampled upon fitting of the molecule to the electron density. In cases of poorly defined electron density, however, ligand fitting can be greatly facilitated if the starting conformation is energetically plausible.

Starting coordinates and restraints from a dictionary generator can be easily checked for validity and robustness by carrying out a round of idealization (*i.e.* refinement without the X-ray term; §S2, Supporting Information) and inspecting the output coordinates (Supplementary Fig. S2). In the main, only minor differences are observed between pre- and post-refinement coordinates, as illustrated for the *phenix.elbow* output (Fig. 4 ▸ *b*). However, even subtle changes such as these can impact on the interpretation of a structure, potentially leading to incorrect assignment of protein–ligand interactions; the devil, as ever, lies in the details. The *Libcheck* output is a notable exception to the general rule, and illustrates how, when supplied with appropriate restraints, a powerful refinement engine can begin to unscramble inaccurate input coordinates (Fig. 4 ▸ *c*). Accurate restraints can thus be a powerful way to correct an errant molecule, although a better result will always be achieved by starting from a high-quality coordinate set.

**Figure 5:**
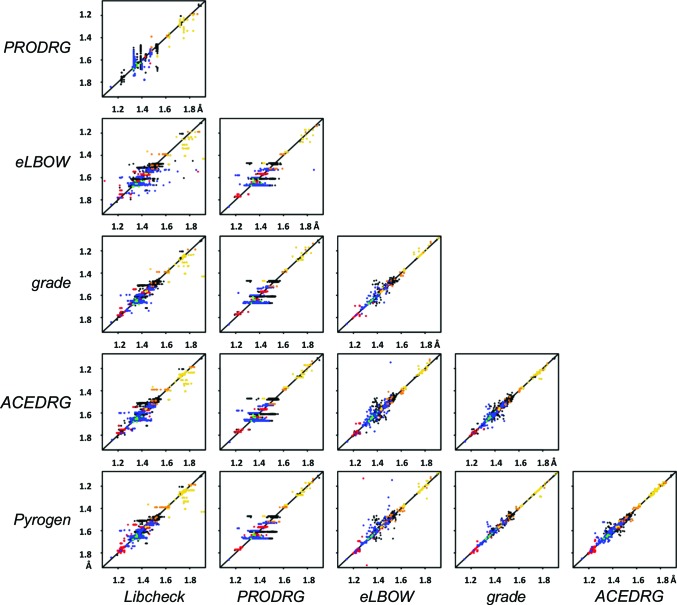
Comparison of bond restraints from selected dictionary generators. Bond-length restraints assigned by program *A* on the vertical axis are plotted in Å against those assigned by program *B* on the horizontal axis. Each matched pair is represented by a dot, where bonds between two C atoms are coloured black and those containing at least one N atom are blue, O atom red, S atom gold, P atom dark orange and halogen (Cl, Br, F or I atom) green. For a more complete description of the methodology underlying this figure, please see §S5 of the Supporting Information.

As illustrated in Figs. 3 ▸ and 4 ▸ in an anecdotal way for the single hypothetical molecule chimerin1, every dictionary generator is different. Analysis of the dictionaries generated for 148 compounds from the CCP4 monomer library shows that this observation holds more generally. A comparison table for bond lengths from dictionaries generated by four different programs (Fig. 5 ▸) shows that the restraints are more similar for certain pairs of programs than for others, reflecting the differences in methodology and data source between the programs. Modern methods (as exemplified here by *ACEDRG*, *grade*, *phenix.elbow* and *Pyrogen*) show greater consistency with one another than older software (exemplified here by *cPRODRG* and *Libcheck*), suggesting a welcome improvement in the accuracy of restraints definition over time.

## 4. Dictionary validation   

Dictionary-generator output should be viewed as a starting point, which will likely evolve during the refinement and model-building process (see, for example, Bax *et al.*, 2017 ▸; Agrawal *et al.*, 2013 ▸; Chan *et al.*, 2015 ▸). One way to check the refined or idealized coordinate geometry (and thereby the dictionary) is to use the Cambridge Crystallographic Data Centre (CCDC) software *Mogul* (Bruno *et al.*, 2004 ▸) to search against the small-molecule data in the CSD. Tools for doing this are now available in *Coot* (Emsley, 2017 ▸) and through the *PDB Validation Server* (Adams *et al.*, 2016 ▸). The version of chimerin1 generated using *ACEDRG* shows overall a good agreement with the data in the CSD, as reflected in the low root-mean-square *Z* (r.m.s.*Z*) values for bond lengths and angles (Table 4 ▸). Two bonds and six angles are, however, flagged as being unusual; the bond and angle outliers with the highest *Z*-score are indicated in Fig. 2 ▸(*e*) (labelled A1 and B1, respectively). Several torsion (or di­hedral) angles are also flagged; T1 in Fig. 2 ▸(*e*) had the largest *d*_min_ value. This torsion angle is quite variable across the output coordinates shown in Fig. 4 ▸(*a*), likely reflecting differences in the conformer/coordinate-generation methods used by the various programs. Interestingly, three angles and four torsions in chimerin1 are not represented in the CSD, and several others are represented by fewer than five examples; a consequence of the novel chemistry of our hypothetical example molecule.

Prior knowledge suggested two further areas for potential manual intervention and editing of the chimerin1 dictionary. These are the following.

(i) The planar definition for the imidazopyridine, which can in some circumstances ‘flex’ over the carbon–nitrogen bond between the two fused rings (*e.g.* in response to the steric constraints of a protein binding site, Julie Tucker & David Buttar, unpublished observation), thus necessitating the definition of this moiety as two conjoined planes. Certain programs (*e.g. grade*) allow the definition of planar groups as a set of smaller intersecting planes, which can be useful in such cases.
(ii) The angles, torsions and planar restraints around the linker N atom, which can have *sp* ^3^ character and thus be nonplanar. As can be seen in Figs. 3 ▸(*e*) and 3 ▸(*g*), *grade* and *Pyrogen* recognize and allow for this nonplanarity at the secondary amine.

In addition to the above-mentioned analyses, it is important to manually sense-check the dictionary and coordinate outputs; does the output molecule make chemical sense? A good fit to the electron density, although important, is insufficient. The molecule should also make sensible interactions with the surrounding protein at the binding site and be appropriately protonated, taking into account the pH of the crystallization buffer and the properties of the binding site (Bax *et al.*, 2017 ▸; Emsley, 2017 ▸).

A number of graphical restraints editors are available (Table 3 ▸) that facilitate the process of checking and adjusting an initial dictionary file where experimental or other information suggest that this may be necessary.

### 4.1. The importance of standard deviations   

**Figure 6:**
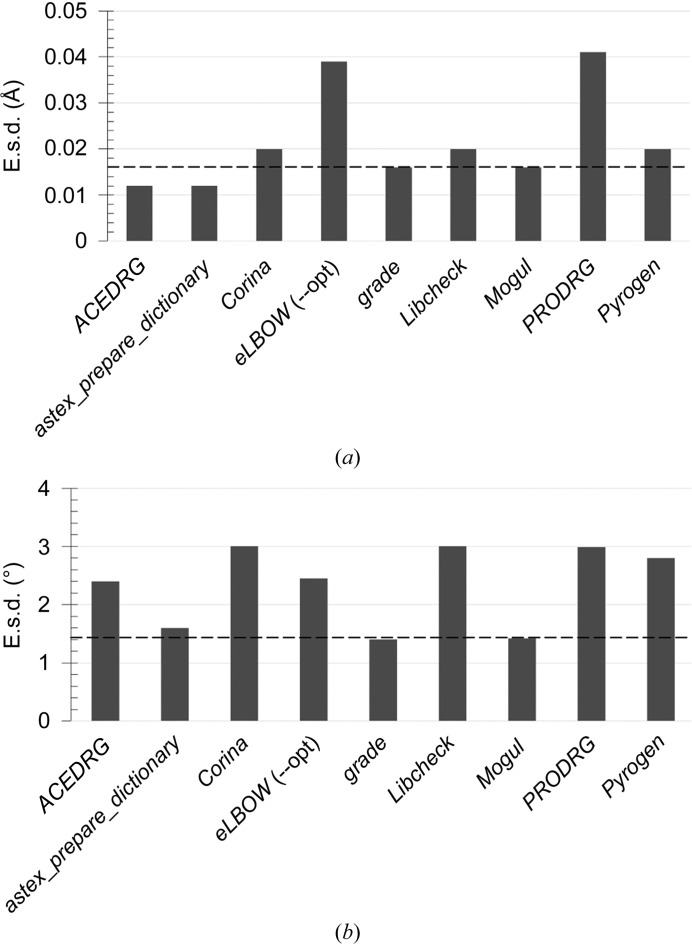
Variation in dictionary-generator standard deviations (e.s.d.) for a selected bond length (Br—C8) (*a*) and bond angle (Br—C8—C7) (*b*) in chimerin1. Atoms are numbered as shown in Fig. 2 ▸(*d*). The standard deviation for all bonds/angles of that type in the CSD obtained using *Mogul* is highlighted as a dashed line.

The standard deviations (σ_target_) for the restraints in chimerin1 varied quite substantially amongst the different output dictionaries, as shown for the carbon–bromine bond (Fig. 6 ▸ *a* and Supplementary Table S2) and a carbon–carbon–bromine angle (Fig. 6 ▸ *b* and Supplementary Table S3). The standard deviation varies from very small (*i.e.* tight restraints) to greater in magnitude than the value returned by *Mogul* for all instances of that bond/angle type in the CSD (*i.e.* loose restraints), and reflects the methodology that each of the dictionary generators uses to derive the standard deviations. Accurate standard deviations are key to achieving well behaved refinement; an inappropriate weight (where weight = 1/σ^2^_target_; equation 3) on a restraint involving a poorly defined atom (*i.e.* one with weak electron density) can completely distort the geometry of the surrounding atoms in the molecule. A significant advantage of using experimentally derived data to define standard deviations is their resultant accuracy, with the exception of those cases where there are few or no experimental observations. In these instances, a suitable value for the standard deviation may be derived from quantum-mechanical calculations (as implemented in *grade*).

## 5. Summary and future directions   

In summary, a number of ligand dictionary generators are now available, with more in development. They support multiple input and output formats, and use a variety of approaches, both empirical and theoretical, to derive restraint information. Each has its own features and limitations, and all will provide a good starting point for further manual intervention and iterative improvement as knowledge of the small-molecule properties within the macromolecular complex become clearer during refinement.

Many of the small molecules for which structures have been solved in complex with a macromolecule are underrepresented in the small-molecule structure databases (Groom *et al.*, 2016 ▸), limiting the availability of experimentally derived restraints. Recent advances in small-molecule crystallization that allow crystals (and their structures) to be generated using small amounts of material (for example, the use of metal–organic frameworks as ‘crystalline sponges’; Inokuma *et al.*, 2013 ▸) suggest that it may be possible, and even desirable, to determine the structures of the small-molecular and macromolecular parts of a complex in parallel, thus helping to fill the gaps in our knowledge that arise from the current limited coverage of chemical space in small-molecule structure databases.

There remain areas for further work, including metals (which present additional challenges owing to their variable coordination and oxidation states), sugars and tautomers, all of which will be covered in more detail by other contributions to these proceedings (Agirre, 2017 ▸; Bax *et al.*, 2017 ▸; Zheng *et al.*, 2017 ▸). Can we aspire to a dictionary generator that ‘works first time, every time’? Such a program would need to take into account the ligand environment, as well as the ligand itself. To conclude, future improvements in dictionary generation will no doubt result, as they have in the past, from continued constructive dialogue between those who use dictionaries and those who write the software that generates them.

## 6. Related literature   

The following reference is cited in the Supporting Information for this article: R Core Team (2015 ▸).

## Figures and Tables

**Table 1:** Atom energy types for three C atoms in the imidazopyridine ring of chimerin1

Atom name†	Atom energy type	Atom energy type description
C3	CR5	Carbon without hydrogen in five-atom ring
C7	C1	Carbon connected to one hydrogen
C8	CR6	Carbon without hydrogen in six-atom ring

† Atoms are numbered as shown in Fig. 2 ▸(*d*).

**Table 2:** Some force fields used in ligand dictionary-generation software

Force field	Full name	Citation	Parametrization†	Usage
MMFF94	Merck Molecular Force Field 94	Halgren (1996 ▸)	Electronic structure calculations	*Pyrogen*, *eLBOW*, *writedict*
AM1	Austin Model 1	Dewar *et al.* (1985 ▸)	Semi-empirical method	*eLBOW*, *grade*
RM1	Recife Model 1	Rocha *et al.* (2006 ▸)	Semi-empirical method	*eLBOW*, *grade*
PM3	Parametrized Model No. 3	Stewart (1989 ▸)	Semi-empirical method	*eLBOW*, *grade*
GROMOS96 43A1	GROningen MOlecular Simulation	Schuler *et al.* (2001 ▸)	Semi-empirical method; limited number of atom types	*PRODRG*

† Semi-empirical methods use theory, approximation and experimental data to speed up calculations.

**Table:** 

Program name	*ACEDRG*	*astex_prepare_dictionary*	*Corina*	*Grade*
Distributor	CCP4	n/a	Molecular Networks	Global Phasing
Latest release	Jan 2016	n/a	Jan 2015	Jul 2014
Input formats	SMILES, PDB, CIF	SMILES, PDB	SMILES	SMILES, Molfile, CIF
Output formats	PDB, CIF	Multiple, including PDB, CIF	PDB, CIF	PDB, CIF, SHELX
Experimental data source(s)	COD (curated)	CSD, Corina	CSD (curated)†	CSD
Force field(s)	None	None	Chem-X‡	AM1/RM1/PM3
Standard deviation source(s)	COD (curated)	CSD (filtered)	CSD (filtered)	CSD
Restraints editor	*JLigand* §	None	None	Edit *REFMAC*
Other features and limitations	Hierarchical atom typing	Proprietary (Astex)	High-quality coordinate generator	Flexible planar definitions. Available through web server.
Citation	Long *et al.* (2017 ▸)	Mooij *et al.* (2006 ▸)¶	Sadowski *et al.* (1994 ▸), Schwab (2010 ▸)	Smart *et al.* (2011 ▸)

**Table:** 

Program name	*eLBOW*	*PRODRG*2	*Pyrogen*	*Writedict*
Distributor	PHENIX	Dundee University	CCP4	OpenEye
Latest release	Oct 2015	Jan 2005	Sep 2016	Oct 2014
Input formats	SMILES, PDB, CIF	PDB, Molfile, sketch, text drawing	SMILES, CIF, sketch	SMILES
Output formats	Multiple, including PDB, CIF	Multiple, including PDB, CIF, CNS, GROMACS	PDB, CIF	PDB, CIF, TOPPAR
Experimental data source(s)	CSD	CSD	CSD, ener_lib.cif	n/a
Force field(s)	Multiple including AM1, MMFF94	GROMOS96 43A1	MMFF94	MMFF94
Standard deviation source(s)	Multiple including CSD	GROMOS force constraints	CSD	Engh & Huber (1991 ▸)
Restraints editor	*REEL*	None	*Coot* restraints editor	None
Other features and limitations	Atom name preservation. Metal coordination.	Limited atom types (no metals). Available through web server. *cPRODRG *within CCP4 distribution accepts SMILES.	Atom name preservation. Tautomer enumeration.	Atom name preservation. Covalent link detection.
Citation	Moriarty *et al.* (2009 ▸)	Schüttelkopf & van Aalten (2004 ▸)	Debreczeni & Emsley (2012 ▸), Emsley & Debreczeni (2012 ▸)	Wlodek *et al.* (2006 ▸)

† Bond lengths and angles are taken from tables (*e.g.* Allen *et al.*, 1987 ▸), which are themselves derived from values in the CSD.

‡ Chem-X molecular modelling software, developed and distributed by Chemical Design Ltd, Oxford, England, 1990.

§ Lebedev *et al.* (2012 ▸).

¶ For further details of methodology, see §S3 in the Supporting Information.

**Table 4:** Example *Mogul* validation summary for chimerin1 Coordinates for chimerin1 were generated using *ACEDRG*, subjected to ten cycles of idealization in *REFMAC*5 and then used as the search query in *Mogul* as described in §§S2 and S4 in the Supporting Information.

Bond lengths		Bond angles	
R.m.s.*Z*	No. with *Z* > 2	R.m.s.*Z*	No. with *Z* > 2
1.04	2 of 23	2.58	6 of 31†

† Three angles gave no hits.
